# Prognostic significance of cell cycle-associated proteins p16, pRB, cyclin D1 and p53 in resected oropharyngeal carcinoma

**DOI:** 10.1186/s40463-018-0298-3

**Published:** 2018-09-06

**Authors:** Michaela Plath, Martina A. Broglie, Diana Förbs, Sandro J. Stoeckli, Wolfram Jochum

**Affiliations:** 1 Department of Otorhinolaryngology, Head and Neck Surgery, Kantonsspital St.Gallen, St. Gallen, Switzerland; 2 Department of Otorhinolaryngology, University Hospital Heidelberg, Heidelberg, Germany; 3 Institute of Pathology, Kantonsspital St. Gallen, Rorschacher Strasse 95, 9007 St. Gallen, Switzerland; 4 Department of Otorhinolaryngology, Head and Neck Surgery, UniversitätsSpital Zürich, Zurich, Switzerland

**Keywords:** Oropharyngeal squamous cell carcinoma, Human papillomavirus, p16, Retinoblastoma protein, Cyclin D1, p53, Prognosis

## Abstract

**Background:**

Human papillomavirus (HPV)-related oropharyngeal squamous cell carcinoma (OPSCC) has an improved outcome and may allow for treatment de-escalation. High-risk HPV (HR-HPV) infection is associated with deregulated expression of the cell cycle-associated proteins p16^INK4^, pRB, cyclin D1 and p53. The objective of this study was to assess cell cycle proteins as potential surrogate markers for HR-HPV DNA testing to identify OPSCC with favorable prognosis after resection.

**Methods:**

Tissue microarray cores of 313 surgically treated OPSCC were stained for p16^INK4a^, pRB, cyclin D1 and p53 using immunohistochemistry. Protein expression was scored as high or low based on the proportion of positive carcinoma cells. Tumor samples were analysed for HR-HPV DNA with polymerase chain reaction-based testing. Associations between cell cycle protein expression and HR-HPV DNA status were evaluated by calculating sensitivity, specificity, predictive values, and diagnostic odds ratios (DOR). Kaplan-Meier and Cox regression analysis were applied to evaluate associations between cell cycle protein expression and patient outcome.

**Results:**

High expression of p16^INK4a^, cyclin D1, pRB and p53 in tumor cells were observed in 51.8%, 51.4%, 41.9% and 33.5% of OPSCC, respectively. HR-HPV DNA positive were 158/313 (50.5%) tumor samples (HPV16: 147, HPV18: 1, HPV33: 5, HPV35: 2, HPV56: 2, and HPV59: 1). P16^INK4a^ showed a higher DOR to predict HR-HPV DNA positivity than pRB, cyclin D1 and p53. Both the p16^INK4a^/pRB and the p16^INK4a^/pRB/cyclin D1/p53 signatures had lower DOR than p16^INK4a^ alone. Improved 5-year overall and disease-specific survival were associated with HR-HPV DNA positivity, high p16^INK4a^, low pRB, low cyclin D1, and low p53 expression. Associations with improved outcome were also observed for the marker combinations high p16^INK4a^/positive HR-HPV DNA, high p16^INK4a^/low pRB and high p16^INK4a^/low pRB/low cyclin D1/low p53. In a multivariate analysis adjusted for age, smoking history, pT and pN category, high p16^INK4a^ expression showed the lowest hazard ratio for death.

**Conclusions:**

High p16^INK4a^ expression is a reliable marker for survival prognostication in surgically treated OPSCC patients. Protein signatures including the pRB, cyclin D1 and p53 proteins do not further increase the prognostic performance of p16^INK4a^ as a single marker.

## Background

High-risk human papillomavirus (HR-HPV) infection is a major cause of oropharyngeal squamous cell carcinoma (OPSCC) [1]. HPV-related OPSCC has better prognosis, irrespective of disease stage [2–4]. Treatment options for patients with early OPSCC include surgical resection with or without adjuvant radio (chemo)therapy or primary radio(chemo)therapy. Identification of the prognostic impact of HPV has lead to the development of risk-adapted staging systems for HPV-related OPSCC, and to the adaptation of the current American Joint Committee on Cancer (AJCC)/Union for International Cancer Control (UICC) TNM staging classification [2–5]. These developments underscore the increasing clinical significance of HPV status in OPSCC und the need for establishing the HPV status of OPSCC prior to treatment initiation.

HR-HPV infection of tumor cells is associated with the expression of the viral E6 and E7 oncoproteins [6]. Both proteins are essential to induce and maintain cellular transformation, due to their interference with cell cycle control, apoptosis, and genomic stability. In addition to other target proteins, E6 promotes the degradation of the p53 protein, which regulates growth arrest and apoptosis after DNA damage. E7 increases degradation of the cellular retinoblastoma protein (pRB), which leads to up-regulation of p16^INK4a^ and down-regulation of cyclin D1. Disruption of both the p53 and pRB pathways promotes S-phase entry. The tumor cell signature of high p16^INK4a^ expression and low/absent expression of cyclin D1, pRB and p53 correlates with HR-HPV infection [7]. Furthermore, deregulated expression of these cell cycle regulators has been linked to outcome prediction in OPSCC. High p16^INK4a^ expression of tumor cells was associated with favorable prognosis and high cyclin D1 and p53 expression of tumor cells was linked to poor outcome [8–19]. However, evaluation of the prognostic significance of deregulated cell cycle regulator expression in OPSCC has been restricted by usage of different immunohistochemical staining methods, scoring systems, and thresholds, and most importantly heterogeneity of patient cohorts with respect to disease stage and treatment modalities.

The objectives of our study were to validate p16^INK4a^ as a prognostic marker in a large cohort of surgically treated OPSCC, and to directly compare the performance of p16^INK4a^ with pRB, cyclin D1 and p53 to predict patient outcome. The second objective was to evaluate the cell cycle proteins p16^INK4a^, pRB, cyclin D1 and p53 as surrogate markers for HR-HPV DNA positivity.

## Methods

### Patient population

The cohort of this study represents a subgroup of a previous reported cohort [20]. Demographic and clinical data were collected by chart review. The patients were categorized into two groups based on treatment: Group 1 was treated with resection alone (*n* = 104; 33.2%), group 2 with resection and adjuvant radio(chemo)therapy (*n* = 209; 66.8%). Pathological staging was performed using the 7th edition of the AJCC/UICC TNM classification system (2010).

### Histological analysis

One representative formalin-fixed paraffin-embedded tissue block of each OPSCC resection specimen was retrieved. Hematoxylin and eosin (H&E) staining of sections was performed using standard techniques. Stained sections underwent histological review to confirm the presence of carcinoma.

### Tissue microarray

Formalin-fixed paraffin-embedded OPSCC tissue samples were used to construct tissue microarrays (TMA). Three cores (diameter 2 mm) with tumor tissue were punched out of each donor block and transferred to the recipient block. Tissue cores from tonsils of non-tumor patients were included as controls. H&E stained TMA sections were microscopically evaluated for the presence of tumor cells.

### Immunohistochemistry

Three-μm thick tissue microarray sections were deparaffinized followed by antigen retrieval (EDTA pH 9.0, 95 °C, 30 min). Stainings were performed on a Leica BOND MAX instrument (Leica) using the Bond Polymer Refine detection kit (Leica) and the following primary antibodies: p16^INK4a^ (clone E6H4, dilution 1:10, 30 min; MTM Laboratories, Heidelberg, Germany), pRB (clone 13A10, dilution 1:100, 15 min; Novocastra), cyclin D1 (clone SP4, dilution 1:50, 30 min; ThermoScientific), and p53 (clone DO-7, dilution 1:150, 30 min; Dako). Marker expression was scored as high or low based on the proportion of positive carcinoma cells (high p16^INKa^: more than 70% of tumor cells; high pRB, cyclin D1, or p53: more than 25% of tumor cell nuclei) [19, 21].

### HPV DNA analysis

Tumor cells were located and marked on H&E stained tissue sections obtained from paraffin blocks of primary OPSCC. Selective dissection of tumor cells was performed from three to five 4 μm thick serial tissue sections of the same paraffin block and genomic DNA was prepared. HPV DNA was detected by polymerase chain reaction (PCR)-based amplification using the L1C1/L1C2 consensus primer set as previously described [22]. PCR products were purified using the QIAGEN PCR purification Kit (QIAGEN) and underwent direct sequencing for HPV typing. Negative and positive controls were included in each HPV PCR run. Beta-globin PCR was performed for samples with negative HPV PCR to test for effective DNA extraction and DNA integrity. Only samples with amplifiable DNA were scored as negative for HPV.

### Statistical analysis

All analyses were performed with the SPSS Statistics 22 software (IBM Corporation, Armonk NY). Demographic and clinicopathological patient characteristics were investigated with descriptive statistics. Associations between cell cycle protein expression and HR-HPV DNA status were analyzed using the chi-square test. Specificity, sensitivity, positive predictive values (PPV), negative predictive values (NPV), and diagnostic odds ratios (DOR) were calculated to evaluate cell cycle proteins and expression signatures as potential surrogate markers for HR-HPV DNA positivity. Overall survival (OS) was defined as time from the date of cancer diagnosis to the date of death, disease-specific survival (DSS) as the time from the date of cancer diagnosis to the date of death from OPSCC. Five-year OS and DSS rates were calculated using the Kaplan-Meier method. Univariate Cox proportional hazard models were used to explore associations between patient characteristics, biomarker results, OS and DSS. Hazard ratios (HR) with 95% confidence intervals were calculated. The Cox proportional hazard model was used for multivariate analysis. *P* values of < 0.05 were considered statistically significant.

## Results

### Patient cohort

Three hundred thirteen patients with surgically treated and histologically confirmed OPSCC were included in the analysis. The clinicopathological characteristics of the patient cohort are shown in Table 1.

**Table 1:** Clinicopathological characteristics of the study population

Characteristic	Entire cohort (*n* = 313)	Resection only group (*n* = 104)	Resection with adjuvant therapy group (*n* = 209)
Mean age (range)	68 (35–101)	68 (47–101)	68 (35–100)
Gender			
Male	230 (73.5%)	71 (68.3%)	159 (76.1%)
Female	83 (26.5%)	33 (31.7%)	50 (23.9%)
Smoking history			
> 10 pack-years	221 (70.6%)	78 (75.0%)	143 (68.4%)
< 10 pack-years	92 (29.4%)	26 (25.0%)	66 (31.6%)
Alcohol intake			
< 3 U/d	164 (52.4%)	52 (50.0%)	112 (53.6%)
> 3 U/d	113 (36.1%)	40 (38.5%)	73 (34.9%)
Missing data	36 (11.5%)	12 (11.5%)	24 (11.5%)
Primary site			
Tonsil	217 (69.3%)	68 (65.4%)	149 (71.3%)
Base of tongue	58(18.5%)	16 (15.4%)	42 (20.1%)
Posterior wall / soft palate	38 (12.2%)	20 (19.2%)	18 (8.6%)
Tumor stage			
T1/T2	263 (84.0%)	96 (92.3%)	167 (79.9%)
T3/T4	50 (16.0%)	8 (7.7%)	42 (20.1%)
Nodal stage			
N0	92 (29.4%)	58 (55.8%)	34 (16.3%)
N+	221 (70.6%)	46 (44.2%)	175 (83.7%)
HR-HPV DNA			
Positive	158 (50.5%)	42 (40.4%)	116 (55.5%)
Negative	155 (49.5%)	62 (59.6%)	93 (44.5%)
5-year overall survival rate	76.1%	69.8%	79.1%
5-year disease-specific survival rate	85.2%	83.1%	86.2%

### Cell cycle protein expression

In non-neoplastic tonsils, scattered epithelial cells showed weak to moderate p16^INK4a^ reactivity. pRB was strongly expressed in the nuclei of basal and parabasal epithelial cells, which also showed weak to moderate nuclear cyclin D1 and p53 staining (data not shown).

**Fig. 1:**
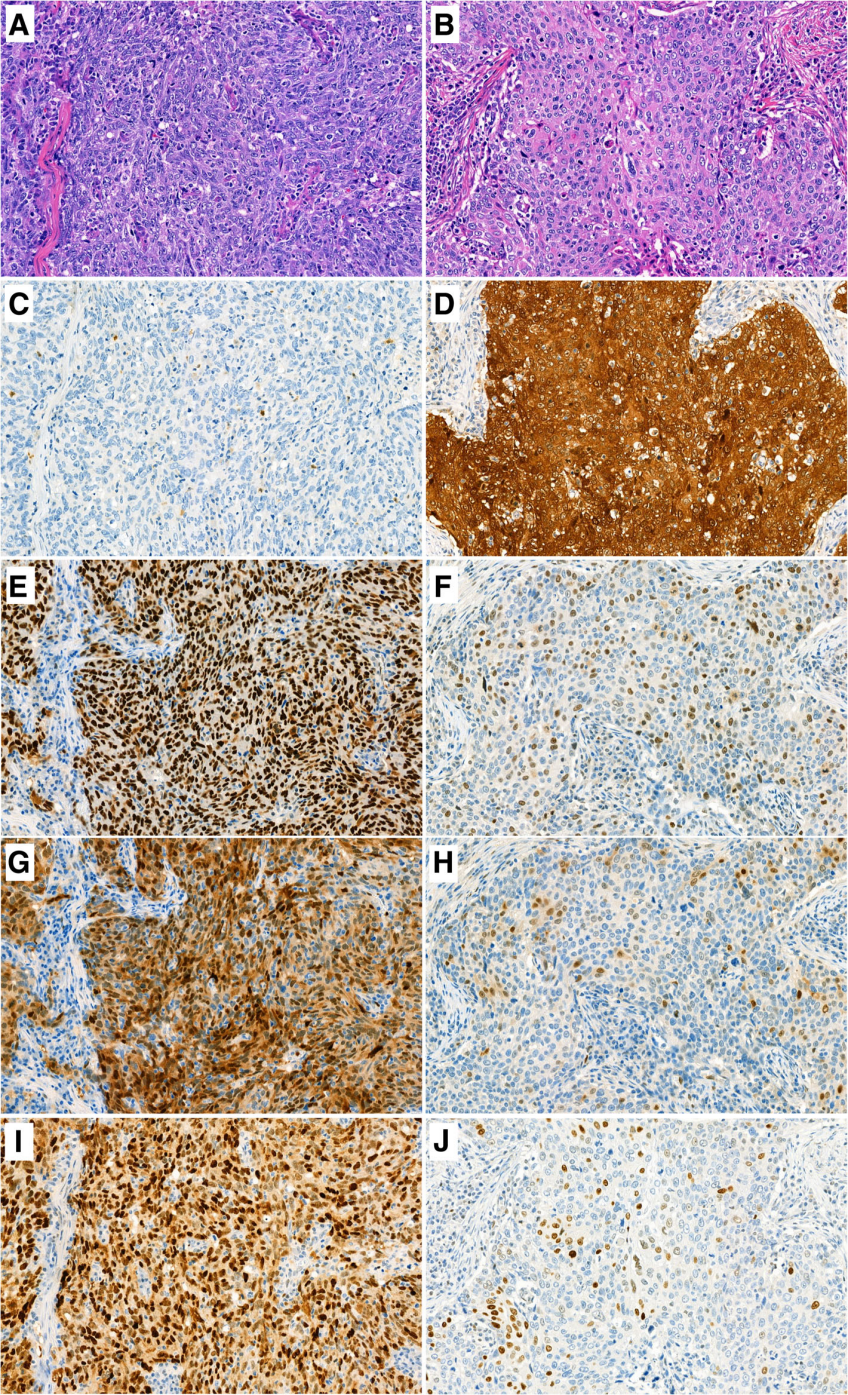
Expression of cell cycle-associated proteins in oropharyngeal squamous cell carcinoma. Histological findings (**a**, **b**) and expression of the p16^INK4a^ (**c**, **d**), pRB (**e**, **f**), cyclin D1 (**g**, **h**) and p53 (**i**, **j**) proteins in representative HR-HPV positive (**b**, **d**, **f**, **h**, **j**) and HR-HPV negative OPSCC (**a**, **c**, **e**, **g**, **i**). Original magnification, 400×

We next evaluated cell cycle protein expression in tissue cores of 313 OPSCC (Fig. 1, Table 2). Strong p16^INK4a^ staining in more than 70% of carcinoma cells was observed in 52.1% of OPSCC. pRB and cyclin D1 reactivity was low (< 25% of tumor cells) or absent in 48.6% and 57.8% of OPSCC, respectively. Nuclear p53 positivity stronger than in tonsillar epithelial cells was observed in 33.5% of tumors. A high p16^INK4a^/low pRB signature of tumor cells was present in 45.4% of OPSCC. Thirty-eight percent of the OPSCC displayed a high p16^INK4a^/low pRB/low cyclin D1/ low p53 signature.

**Table 2:** Association between cell cycle protein expression and HR-HPV DNA status

Cell cycle protein	Entire cohort	HR-HPV DNA negative	HR-HPV DNA positive			Pearson Chi-square	Sensitivity (%)	Specificity (%)	PPV (%)	NPV (%)	DOR
	(*n* = 313)	(*n* = 155)	All (*n* = 158)	HPV16 (*n* = 147)	Non-HPV16 (*n* = 11)	*p* value					
p16^INK4a^						< 0.001	91.8	89.0	89.5	91.4	90.3
Low	151 (48.2%)	138 (89.0%)	13 (8.2%)	13 (8.8%)	0 (0%)						
High	162 (51.8%)	17 (11.0%)	145 (91.8%)	134 (91.2%)	11 (100.0%)						
pRB						< 0.001	82.9	86.5	86.2	83.2	31.1
Low	152 (48.6%)	21 (13.5%)	131 (82.9%)	121 (82.3%)	10 (91.0%)						
High	161 (51.4%)	134 (86.5%)	27 (17.1%)	26 (17.7%)	1 (9.0%)						
Cyclin D1						< 0.001	84.2	68.4	73.1	80.9	11.5
Low	181 (57.8%)	48 (31%)	133 (84.2%)	124 (84.3%)	9 (81.8%)						
High	132 (42.2%)	107 (69%)	25 (15.8%)	23 (15.7%)	2 (18.2%)						
p53						< 0.001	85.4	53.9	64.9	78.1	6.8
Low	208 (66.5%)	73 (47.1%)	135 (85.4%)	127 (86.4%)	8 (72.7%)						
High	105 (33.5%)	82 (52.9%)	23 (14.6%)	20 (13.6%)	3 (27.3%)						
p16^INK4a^/pRB						< 0.001	82.9	92.9	92.3	84.2	63.8
High/low	142 (45.4%)	11 (7.1%)	131 (82.9%)	121 (82.3%)	10 (90.9%)						
Other	171 (54.6%)	144 (92.9%)	27 (17.1%)	26 (17.7%)	1 (9.1%)						
p16 ^INK4a^/pRB/ cyclin D1/p53						< 0.001	68.4	93.5	91.5	74.4	30.9
High/low/low/low	118 (37.7%)	10 (6.5%)	108 (68.4%)	102 (69.4%)	6 (54.6%)						
Other	195 (62.3%)	145 (93.5%)	50 (31.6%)	45 (30.6%)	5 (45.4%)						

*PPV* positive predictive value, *NPV* negative predictive value (%); and *DOR* diagnostic odds ratio. Non-HPV16 group included tumors positive for HPV18, HPV33, HPV35, HPV56, or HPV59

### Association of cell cycle protein expression with HR-HPV DNA

HPV DNA testing was conclusive in all 313 OPSCC. 158 (50.5%) tumors were HR-HPV DNA positive (HPV16: 147, HPV18: 1, HPV33: 5, HPV35: 2, HPV56: 2, and HPV59: 1). HR-HPV DNA positive OPSCC were positive for p16^INK4a^, pRB, cyclin D1and p53 in 91.8%, 17.1%, 15.8%, and 14.6%, respectively (Table 2). There was a strong association between HR-HPV DNA positivity and high p16^INK4a^ expression. In addition, HR-HPV DNA positivity was also correlated with low pRB, low cyclin D1 and low p53 expression. There was also a strong association between HR-HPV DNA positivity and both the 2-marker signature high p16^INK4a^/low pRB and the 4-marker signature high p16^INK4a^/low pRB/low cyclin D1/low p53. High p16^INK4a^ expression alone displayed the highest sensitivity value for HR-HPV DNA positivity. In contrast, the 4-marker signature showed the highest specificity value. To directly compare the performance of individual cell cycle proteins and the two marker signatures to detect HR-HPV DNA positive OPSCC, diagnostic odds ratios (DOR) were calculated. The value of a DOR ranges from 0 to infinity, with higher values indicating better discriminatory test performance. The largest DOR value was observed for high p16^INK4a^ expression alone (Table 2).

### Association of cell cycle protein expression with survival after resection

**Fig. 2:**
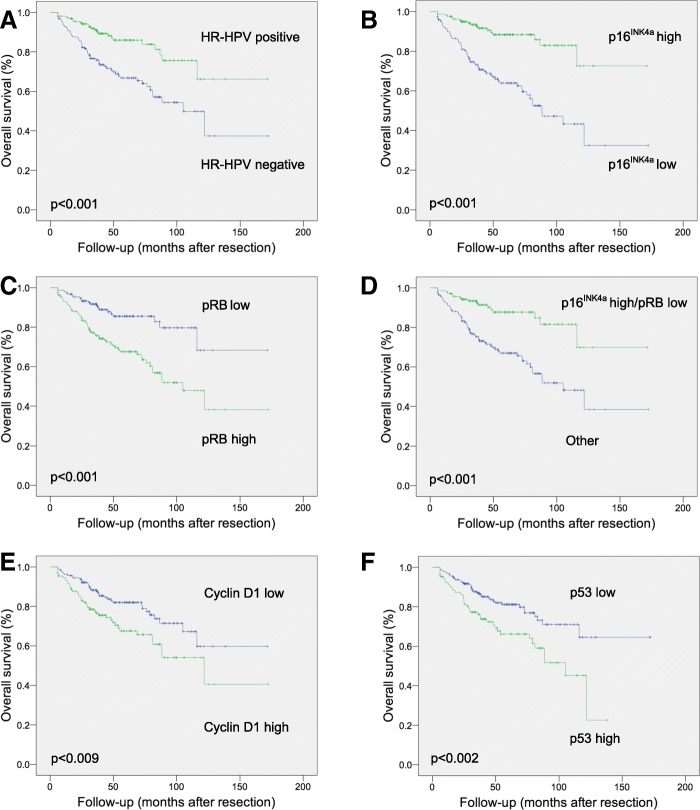
Overall survival after resection in patients with oropharyngeal squamous cell carcinoma. Kaplan-Meier analyses revealed associations between improved survival and HR-HPV DNA positivity (**a**), high p16^INK4a^ (**b**), low pRB (**c**), high p16^INK4a^/low pRB signature (**d**), low cyclin D1 (**e**), and low p53 (**f**)

The mean follow-up period of the cohort was 119 months (range 6–172 months). The 5-year OS and DSS rates were 76.1% and 85.2%, respectively. Kaplan-Meier analysis demonstrated that both improved OS and DSS were associated with HR-HPV DNA positivity, and high p16^INK4a^, low pRB, low cyclin D1 and low p53 expression of tumor cells (Fig. 2, Table 3).

**Table 3:** Univariate analysis of overall and disease-specific survival for patients with OPSCC patients treated with resection

*N* = 313	5-year overall survival				5-year disease-specific survival			
Parameter	%	*P* value	HR (95% CI)	*P* value	%	*P* value	HR (95% CI)	*P* value
Age (> 65 y vs. < 65 y)	75.6/77.1	0.83	1.05 (0.66–1.68)	0.83	85.3/84.9	0.88	1.05 (0.56–1.99)	0.88
Smoking history (> 10 py vs. < 10 py)	70.9/88.5	0.002	2.53 (1.37–4.69)	0.003	80.8/95.8	0.003	4.19 (1.49–11.77)	0.006
Tumor stage (T3/T4 vs. T1/T2)	64.7/78.5	0.15	1.48 (0.87–2.55)	0.15	74.6/87.4	0.023	2.15 (1.09–4.21)	0.03
Nodal stage (N+ vs. N0)	73.5/82.0	0.31	1.3 (0.78–2.16)	0.31	83.2/90.2	0.14	1.77 (0.82–3.84)	0.15
HR-HPV DNA (Positive vs. negative)	85.9/66.8	< 0.001	0.39 (0.24–0.64)	< 0.001	91.6/78.9	< 0.001	0.30 (0.15–0.62)	0.001
p16^INK4a^ (High vs. low)	89.6/64.5	< 0.001	0.29 (0.15–0.44)	< 0.001	93.2/80.9	< 0.001	0.21 (0.1–0.46)	< 0.001
pRB (High vs. low)	67.6/85.5	< 0.001	2.68 (1.63–4.43)	< 0.001	78.8/92.2	< 0.001	3.56 (1.70–7.47)	0.001
Cyclin D1 (High vs. low)	67.6/82.0	0.009	1,79 (1.15–2.80)	0.01	81.9/87.7	0.068	1.76 (0.95–3.25)	0.07
p53 (High vs. low)	66.2/81.2	0.002	1.99 (1.27–3.10)	0.002	79.1/88.2	0.053	1.82 (0.98–3.36)	0.06
High p16^INK4a^/HR-HPV DNA positive vs. others	85.9/66.8	< 0.001	0.39 (0.24–0.64)	< 0.001	91.6/78.9	< 0.001	0.30 (0.15–0.62)	0.001
High p16^INK4a^/low pRB vs. others	87.7/67.0	< 0.001	0.32(0.18–0.54)	< 0.001	93.2/78.7	< 0.001	0.23 (0.10–0.52)	< 0.001
High p16^INK4a^/low pRB/low cyclin D1/low p53 vs. others	88.3/69.9	< 0.001	0.33 (0.18–0.59)	< 0.001	94.1/80.0	< 0.001	0.21 (0.08–0.55)	0.001

Abbreviations: *HR* hazard ratio for death, *CI* confidence interval; *py* pack-year. Log-rank test. *p* < 0.05 was considered statistically significant

Univariate Cox regression analysis revealed reduced overall risks of death for OPSCC with HR-HPV DNA positivity and high p16^INK4a^ expression (Table 3). In contrast, hazard ratios (HR) were increased for OPSCC with high pRB, high cyclin D1, or high p53 expression. The combined parameters high p16^INK4a^/positive HR-HPV DNA, high p16^INK4a^/low pRB, and high p16^INK4a^/low pRB/low cyclin D1/low p53 were also associated with reduced overall risks of death. Comparable results were obtained for the risk of disease-specific death, which was evaluated as an additional outcome parameter (Table 3). We also used Cox proportional hazard models adjusted for age, smoking history, pT stage and pN stage to evaluate and compare the prognostic impact of the various parameters. Parameters retained prognostic significance in multivariate analyses except high cyclin D1 and p53 expression (Table 4).

**Table 4:** Multivariate analysis of overall and disease-specific survival for patients with OPSCC patients treated with resection

	5-year overall survival		5-year disease-specific survival	
Parameter	HR (95% CI)	*p* value	HR (95% CI)	*p* value
HR-HPV DNA (Positive vs. negative)	0.46 (0.26–0.81)	0.007	0.41 (0.18–0.90)	0.027
p16^INK4a^ (High vs. low)	0.23 (0.16–0.57)	< 0.001	0.22 (0.09–0.52)	0.001
pRB (High vs. low)	2.54 (1.46–4.41)	0.001	3.20 (1.46–6.99)	0.004
Cyclin D1 (High vs. low)	1.59 (0–2.55)	0.053	1.55 (0.82–2.94)	0.18
p53 (High vs. low)	1.79 (1.12–2.86)	0.015	1.52 (0.80–2.87)	0.20
High p16^INK4a^/HR-HPV DNA positive vs. others	0.30 (0.16–0.57)	< 0.001	0.26 (0.10–0.64)	0.004
High p16^INK4a^/low pRB vs. others	0.32 (0.18–0.45)	< 0.001	0.25 (0.11–0.60)	0.002
High p16^INK4a^/low pRB/low cyclin D1/low p53 vs. others	0.36 (0.19–0.38)	0.001	0.25 (0.09–0.65)	0.005

Abbreviations: *HR* hazard ratio for death, *CI* confidence interval; *P* < 0.05 was considered statistically significant

We also performed subgroup analyses for OPSCC patients with or without adjuvant treatment after resection. For both subgroups, similar associations between the various parameters and patient outcome were observed as for the entire cohort (data not shown).

## Discussion

Among patients with OPSCC, HR-HPV infection of tumor cells is associated with favorable prognosis, and therefore may identify patients who benefit from treatment de-escalation. Various methods are available for HR-HPV testing. HR-HPV E6/E7 messenger RNA (mRNA) expression demonstrated by reverse transcriptase-PCR or RNA in situ hybridization (ISH) is considered the most specific marker for biologically relevant HPV infection because it identifies transcriptionally active HPV infection and indicates the translation of the viral E6 and E7 oncoproteins [23]. In addition, HR-HPV DNA can be detected by polymerase chain reaction (PCR)-based assays or ISH techniques with HPV type-specific probes [24]. HPV E6 and E7 oncoproteins induce p16^INK4a^ protein overexpression and suppress the expression of the cell cycle proteins pRB, cyclin D1 and p53 in infected cells [6]. Recent studies have shown that the combined detection of HR-HPV DNA (by PCR or ISH) and p16^INK4a^ protein overexpression identifies transcriptionally active HR-HPV infection with specificity and sensitivity comparable to those of E6/E7 mRNA expression [14, 23, 25]. However, the availability of molecular detection methods for HR-HPV DNA or HR-HPV E6/E7 mRNA is limited. In contrast, immunohistochemistry for protein expression analysis is broadly available and therefore may be used as a fast and inexpensive methodology to assess tumor cells for HR-HPV-related protein deregulation in the clinical setting. The 8th edition TNM classification for head and neck cancer distinguishes between OPSCC with and without HPV association, and recommends p16^INK4a^ immunostaining for the identification of tumors with positive HPV status, based on the conclusion that p16^INK4a^ is a reliable surrogate marker for oncologically relevant HPV infection [26]. In HR-HPV infected tumor cells, increased p16^INK4a^ expression is a direct consequence of pRB inactivation by the HPV oncoprotein E7, which induces degradation through the ubiquitin-proteasome pathway [27]. However, using p16^INK4a^ expression alone as a biomarker for positive HR-HPV status may have limitations due to HR-HPV-independent mechanisms of up-regulation.

In our study, we aimed to evaluate pRB, cyclin D1 and p53 as alternative marker for HR-HPV DNA positivity, and to address the question whether protein signatures including pRB, cyclin D1 and p53 may increase the diagnostic performance of p16^INK4a^ alone. In accordance with other studies, we observed that HPV-associated OPSCC are characterized by strong p16^INK4a^ expression and low or absent expression of pRB, cyclin D1, und p53 [7, 18, 19]. Among the four cell cycle-associated proteins, high p16^INK4a^ staining showed the strongest association with HR-HPV DNA positivity when evaluated as a single marker (sensitivity 92%, specificity 88%). The associations between low/absent expression of pRB, cyclin D1, or p53 alone and HR-HPV DNA positivity were significantly lower. In comparison to high p16^INK4a^ expression alone, we observed higher specificity values for the 2-marker signature high p16^INK4a^/low pRB and the 4-marker signature p16^INK4a^/pRB/cyclin D1/p53, but reduced sensitivity values. Using the diagnostic odds ratio as a performance parameter, high p16^INK4a^ expression alone was the best immunohistochemical marker for HR-HPV DNA positivity. Our findings confirm p16^INK4a^ as a reliable surrogate marker for HR-HPV infection in OPSCC. They also indicate that signatures including the pRB, cyclin D1 and p53 proteins do not significantly increase the diagnostic performance of p16^INK4a^ as a single marker.

Treatment options for patients with early OPSCC include surgical resection with or without adjuvant radio(chemo)therapy, or primary radio(chemo)therapy. In addition to its role in tumorigenesis, HR-HPV infection identifies OPSCC patients with improved survival who may benefit from treatment de-escalation [10, 20, 28]. Previous studies have shown that HR-HPV DNA detection alone, the combined detection of HR-HPV DNA and p16^INK4a^ protein, p16^INK4a^protein expression alone, or HR-HPV E6/E7mRNA expression identify OPSCC patients with favorable prognosis [9–12, 14, 15]. Furthermore, poor prognosis has been linked to high cyclin D1 and p53 expression of tumor cells [8, 13, 16–18]. In the majority of theses studies, association analysis of the various markers with survival was confounded by treatment heterogeneity of cohorts and/or limited patient numbers. Only few previous studies have investigated cohorts of surgically treated OPSCC [18, 29–31]. In the current study, we analyzed the prognostic significance of cell cycle protein expression in a large cohort of surgically treated OPSCC patients from the same geographic region [20]. Univariate analysis revealed that improved outcome after resection (both 5-year OS and DSS) were associated with high p16^INK4a^, low pRB, low cyclin D1, and low p53 expression. Furthermore, HR-HPV DNA positivity and the combined parameters high p16^INK4a^/positive HR-HPV DNA, high p16^INK4a^/low pRB, and high p16^INK4a^/low pRB/low cyclin D1/low p53 were also associated with reduced risks of death. Parameters retained prognostic significance in multivariate analyses except high cyclin D1 and p53 expression. Our results confirm the findings of previous studies and validate them specifically for surgically treated OPSCC patients [8–19, 23]. They indicate that high p16^INK4a^ expression provides comparable prognostic information as HR-HPV DNA positivity in surgically treated OPSCC patients and may therefore serve as a reliable surrogate marker for improved prognosis, if HR-HPV DNA testing is not available.

Limitations of our study include that cell cycle protein expression analyses were performed on TMA cores that may not be representative of the whole tumor. However, various studies have shown excellent concordance between TMA spots and whole tissue sections in immunohistochemical analyses of multiple tumor types. Furthermore, each tumor was represented by three tissue cores in our study to increase concordance. Two individuals blinded for HR-HPV DNA results and clinical outcome information scored all immunohistochemical stainings. However, digital image analysis may have increased standardization of scoring. We used HR-HPV DNA positivity as the gold standard to evaluate cell cycle proteins as potential surrogate markers for HR-HPV infection. However, HR-HPV E6/E7 mRNA detection is considered the best marker for biologically relevant HR-HPV infection. Further studies are necessary to directly compare the performance of p16^INK4a^ expression and HR-HPV E6/E7 mRNA detection in surgically treated OPSCC.

## Conclusion

In conclusion, our results show that immunohistochemical demonstration of high p16^INK4a^ expression in tumor cells is a reliable tool for survival prognostication in surgically treated OPSCC patients. Protein signatures including the pRB, cyclin D1 and p53 proteins do not further increase the prognostic and diagnostic performance of p16^INK4a^ as a single marker. Therefore, p16^INK4a^ may be used to select OPSCC patients with improved prognosis for treatment de-escalation, especially in the absence of HR-HPV DNA and HR-HPV E6/E7 mRNA testing.

## Abbreviations

DSS: Disease-specific survival
HPV: Human papillomavirus
HR: Hazard ratio
NPV: Negative predictive value
OPSCC: Oropharyngeal squamous cells carcinoma
OS: Overall survival
PCR: Polymerase chain reaction
PPV: Positive predictive value
TMA: Tissue microarray
